# Editorial: Mitochondrial function and dysfunction in pathogenic fungi

**DOI:** 10.3389/fphys.2024.1506684

**Published:** 2024-10-29

**Authors:** Rosana Alves, Campbell W. Gourlay

**Affiliations:** ^1^ Centre of Molecular and Environmental Biology (CBMA), University of Minho, Braga, Portugal; ^2^ Kent Fungal Group, School of Natural Sciences, University of Kent, Canterbury, United Kingdom

**Keywords:** fungal pathogenesis, mitochondria, metabolism, stress responses, fungal physiology, antifungals

## Introduction

Mitochondria play central physiological roles in most eukaryotic cells. In pathogenic fungi, mitochondrial function is critically important during host-pathogen interactions, allowing either adaptation to changing environments or detoxification of antifungal drugs. This Research Topic compiles a series of manuscripts investigating mitochondrial aspects of both human- and phyto-pathogenic fungi. The insights gained from these studies are expected to be broadly applicable across diverse fungal species. By integrating both research domains, this compilation not only provides a more holistic understanding of how mitochondrial functionality and dysfunctionality shapes fungal pathogenicity, but also fosters cross-disciplinary approaches that may reveal innovative solutions to combat fungal diseases.

## Human pathogenic fungi

Human fungal infections caused by species such as *Candida*, *Aspergillus*, and *Cryptococcus*, are responsible for a substantial burden of disease worldwide. The World Health Organization (WHO) has recently included all these species on a list of priority fungal pathogens, highlighting the critical need for improved diagnostics, treatments, and research into antifungal resistance (World Health Organization, 2022). This initiative reflects a global effort to prioritize fungal pathogens based on their public health impact and unmet research needs. We have received manuscripts covering these three medically relevant genera. By highlighting the essential roles of mitochondrial functions in fungal pathogenicity, drug resistance, and survival strategies, these studies support an emerging model in which mitochondrial activity drives the physiological adaptations necessary for fungal persistence under antifungal and host-induced stresses.

*Candida albicans*, one of the top-priority pathogens identified by the WHO, is a commensal yeast that can become pathogenic in immunocompromised individuals. *C. albicans* cells can transition between various ploidy states, including diploid (Jones et al., 2004), haploid (Hickman et al., 2013), tetraploid (Lockhart et al., 2003) and aneuploid (Janbon et al., 1998; Selmecki et al., 2006). How these states influence the pathogenicity of *C. albicans* has been a subject of intense research. Here, Boone et al. investigate the effects of *C. albicans* ploidy and mating type locus (MTL) on the antifungal activity of farnesol. Although farnesol has been known for targeting actively respiring mitochondria (Machida et al., 1998), its precise mechanism of action remains elusive. The authors tested their hypotheses based on the structural similarity between farnesol and the farnesylated portion of the *MTL*a pheromone. The study revealed that heterozygous MTLa/α diploids exhibited greater tolerance to farnesol compared to haploid strains. The data is consistent with the idea that mitochondrial function, influenced by MTL type and potentially other factors like energy availability and oxygen status, is crucial for how *C. albicans* and other pathogenic fungi tolerate the otherwise lethal effects of farnesol. Understanding these interactions between mitochondrial dynamics and farnesol resistance could open new avenues for targeted antifungal therapies.

*Aspergillus fumigatus,* another priority pathogen, is a ubiquitous environmental mould that poses significant risks to critically ill and immunocompromised patients. This pathogen can cause severe invasive infections in the respiratory system, such as invasive aspergillosis, and has the potential to disseminate to other organs, including the central nervous system and eyes. This Research Topic received two manuscripts focusing on *A*. *fumigatus*.

One of those studies is presented by Zhou et al., who examined GemA, a mitochondrial GTPase homologous to the yeast Gem1 (Frederick et al., 2004). The study demonstrated that GemA is essential for maintaining mitochondrial morphology and membrane potential. Disruption of GemA in *A*. *fumigatus* resulted in abnormal mitochondrial structures, increased resistance to azoles and terbinafine, and altered drug efflux pump expression. The *gemA* mutant also exhibited compromised cell wall integrity and reduced virulence in a *Galleria mellonella* infection model. These findings are consistent with a key function of mitochondria in ensuring cell wall functions, with direct implications in drug resistance and virulence.

The second study, introduced by Yi et al., explored the antifungal properties of benzyl isothiocyanate (BITC) for treating *A*. *fumigatus* keratitis, a serious eye infection. Their work, using both *in vitro* and *in vivo* models of fungal keratitis, demonstrated that BITC damages fungal cells in a concentration-dependent manner by targeting cell membranes and mitochondria, preventing fungal adhesion, and disrupting biofilms. BITC not only reduced fungal load but also inhibited the inflammatory response mediated by the Mincle signaling pathway, offering a promising therapeutic approach for *A*. *fumigatus* keratitis. The efficacy of BITC might represent a significant advance for the treatment of this specific infection, especially when considering the limited range of existing antifungal therapies.

The third and last priority human fungal pathogen discussed in this Research Topic is *C. neoformans*, also known as an opportunistic environmental yeast. Inhalation of *C*. *neoformans* cells from the environment can lead to infection by initially affecting the lungs (cryptococcosis) and further spreading into the blood (cryptococcaemia) and central nervous system (cryptococcal meningitis). The study conducted by Telzrow et al. highlights how mitochondrial function influences cell wall homeostasis and stress responses, contributing to microbial persistence under antimicrobial and host-induced stress. The authors investigated the role of the *C*. *neoformans* Mar1 protein in regulating cell wall homeostasis and stress responses. Mar1 was found to be highly enriched in mitochondria and crucial for proper mitochondrial function. A mutant strain disrupted in Mar1 showed impaired growth under certain stress conditions, altered ATP levels, and changes in mitochondrial morphology. Additionally, the *mar1* mutant displayed increased fluconazole tolerance, which correlated with reduced mitochondrial activity. As we previously discussed, this result is also consistent with the observations made by Zhou et al. for the *A*. *fumigatus gemA* mutant*.* While several mitochondrial mutants have been reported to display increased susceptibility to antifungal drugs, some recent studies have showed that mitochondrial dysfunction can indeed trigger the opposite, in distinct human fungal pathogens (Alves et al., 2020; Duvenage et al., 2019; Satish et al., 2019). The effects of specific mitochondrial mutations on cell wall structure, and consequently on drug resistance or tolerance, remain difficult to predict. Further research is needed to clarify the molecular mechanisms linking mitochondrial function to cell wall biogenesis and how that might impact the mode of action of different classes of antifungals.

## Phytopathogenic fungi

Beyond human health, fungal pathogens pose a serious threat to global agriculture. Crops such as maize, wheat, and rice, which form the backbone of global security, are susceptible to devasting fungal diseases like rusts, blights, and mildews. Such example includes the cryptic *S. reilianum* f. sp. *zeae*, the causative agent of head smut disease in maize (Schirawski et al., 2005). The study presented by Mendoza et al. highlights intriguing aspects of mitochondrial genetic diversity in *Sporisorium reilianum*, offering new insights into mitochondrial function in phytopathogenic fungi. The authors analyzed whole-genome sequencing data from samples collected globally, uncovering unique mitochondrial sequences in strains from China. These sequences are closely related to those in other pathogenic fungi such as *Sporisorium scitamineum* and *Ustilago bromivora*, suggesting potential horizontal gene transfer or mitochondrial recombination events during the evolutionary history of basidiomycetes. This discovery not only sheds light on mitochondrial dynamics but also opens avenues for developing diagnostic tools based on mitochondrial polymorphisms, advancing our understanding of genetic diversity and mitochondrial roles in fungal pathogenesis.

## Mitochondria as a promising source for antifungal drug targets

The increasing incidence of fungal infections and the emergence of drug-resistant strains underscore the pressing need for new antifungal agents. Current treatments are limited, often toxic, and increasingly ineffective due to resistance. Moreover, climate change is exacerbating the spread and severity of fungal infections, as warming temperatures and shifting ecosystems create more favorable conditions for pathogenic fungi. The overuse of agricultural fungicides is also contributing to cross-resistance to medical antifungals. All studies presented in this Research Topic offer additional evidence that mitochondrial functions and metabolic pathways present promising targets for new antifungal drugs. By disrupting these critical processes, it may be possible to design treatments that are both more effective and less likely to induce resistance, addressing the dual challenges of climate change and antifungal overuse. Additionally, mitochondrial genomes (or mitogenomes) were also highlighted as promising diagnostic tools for the identification and discrimination of cryptic species. Indeed, reliable diagnostic tools are critical for early detection and successful treatment of fungal infections.
